# On the Origin of the Functional Architecture of the Cortex

**DOI:** 10.1371/journal.pone.0000251

**Published:** 2007-02-28

**Authors:** Dario L. Ringach

**Affiliations:** Department of Psychology and Neurobiology, University of California Los Angeles, Los Angeles, California, United States of America; Harvard University, United States of America

## Abstract

The basic structure of receptive fields and functional maps in primary visual cortex is established without exposure to normal sensory experience and before the onset of the critical period. How the brain wires these circuits in the early stages of development remains unknown. Possible explanations include activity-dependent mechanisms driven by spontaneous activity in the retina and thalamus, and molecular guidance orchestrating thalamo-cortical connections on a fine spatial scale. Here I propose an alternative hypothesis: the blueprint for receptive fields, feature maps, and their inter-relationships may reside in the layout of the retinal ganglion cell mosaics along with a simple statistical connectivity scheme dictating the wiring between thalamus and cortex. The model is shown to account for a number of experimental findings, including the relationship between retinotopy, orientation maps, spatial frequency maps and cytochrome oxidase patches. The theory's simplicity, explanatory and predictive power makes it a serious candidate for the origin of the functional architecture of primary visual cortex.

## Introduction

In tangential electrode penetrations across primary visual cortex one often observes systematic changes in the receptive field properties of neurons, such as their preferences for orientation and ocular dominance. Vertical electrode penetrations, in contrast, encounter cells sharing similar response properties [1]–[4]. The full two-dimensional structure of these ‘functional maps’ has been best visualized using intrinsic optical imaging [5], [6]. Using this technique, maps of orientation, ocular dominance, spatial frequency, retinotopy, and color, have been obtained in the early visual cortex of various species [5]–[23]. *In-vivo*, two-photon microscopy, is now yielding a first look at the organization of functional maps with cellular resolution [24], [25]. Despite years of research onto the columnar architecture of the cortex there are many important questions that remain unanswered, including the actual origin of the maps and their shapes, the reasons underlying their mutual relationships and, above all, their functional significance in normal visual processing [26]–[28].

Here I advance a theory that shows promise in explaining the development of simple-cell receptive field and feature maps in cat primary visual cortex [29], [30]. The cat was chosen to develop these ideas because of the wealth of available data in this species. First, data on orientation maps, ocular dominance and spatial frequency columns in cat have been described and analyzed in detail in a number of independent laboratories [7], [10]–[12], [14], [17], [31]–[42]. Second, the optics, anatomy, and electrophysiology of cells in the cat early visual pathway, both in the adult and during development, have been well characterized (see [43], [44] for reviews). Third, the statistics of monosynaptic connectivity between the thalamus and layer 4 in the adult cortex are, so far, available only in the cat [45], [46]. Finally, the spatial statistics of the retinal ganglion cell mosaics in cat have been carefully measured and rigorously modeled [47]–[51].

In the kitten, orientation columns and simple-cell structure are all present from the very beginning, as soon as electrophysiological recordings from cortical cells are feasible. Hubel and Wiesel (1963) first demonstrated that kittens lacking visual experience have cells that are both tuned for orientation and cluster according to their orientation preferences [52]. The first cortical responses are observed in the input recipient layers 4 and 6 and nearly 90% of the cells are dominated by the contralateral eye [36], [53]–[55]. These receptive fields tend to have a single OFF subregion. Neurons with a single ON subregion and others showing the classical simple-cell arrangement with ON and OFF subregions side-by-side appear later [53]. Complex cells, defined by overlapping ON/OFF responses, are not very numerous in these initial stages of development [53] (but see Hubel and Wiesel (1963)). These observations seem at odds with models that posit a competition between ON/OFF cells where one would expect a refinement of simple-cell structure from an initial state of substantial ON/OFF overlap [56], [57]. Furthermore, as pointed out by Crair et al (1998), the fact that segregation of ocular dominance columns develops from an initial condition of strong contralateral dominance presents yet another challenge for developmental models. These investigators have suggested that the early development of the contralateral input sets up the template for the orientation map and that the ipsilateral eye goes ‘along for the ride’. How the ipsilateral input can ‘go along for the ride’ and, at the same time, overcome the initial contralateral dominance to establish ocular-dominance columns is unknown.

Theoretical approaches to cortical map formation based on self-organization and symmetry-breaking normally assume initially random, or disordered, maps [58]–[67]. However, we now know that salient features of the adult cortical organization, including the sub-region segregation of simple cells, orientation, and ocular dominance maps, manifest themselves at the earliest stages of cortical development, before the onset of the critical period. Thus, to fully understand cortical map formation we need to answer the following two separate questions [36], [58], [59], [68]. *Initial map establishment*: What are the factors that determine the structure of maps at the earliest stages of development before the onset of the critical period? *Map maintenance and plasticity*: To what extent do activity-dependent developmental processes maintain, modify, or refine, this initial state? The relative contributions of these two stages of development can be properly assessed only after clear descriptions of both processes are obtained.

Here I focus solely on the first of these questions. How exactly are the initial cortical maps established? Two main hypotheses have been considered so far: (*a*) Correlation-based mechanisms relying on the pattern of spontaneous activity in the retina and the LGN [59], [69]–[73], and (*b*) molecular guidance directing the developing thalamocortical projections into forming the desired maps [68], [74]–[78]. The present study elaborates on an alternative hypothesis: the blueprint for the formation of simple-cells receptive fields in layer 4, the feature maps in the cortex, and many of their inter-relationships, may reside in the layout of the retinal ganglion cell mosaics along with a simple statistical connectivity scheme between the thalamus and the cortex [29], [30]. In what follows we refer to this idea simply as the ‘statistical connectivity’ model.

**Figure 1:**
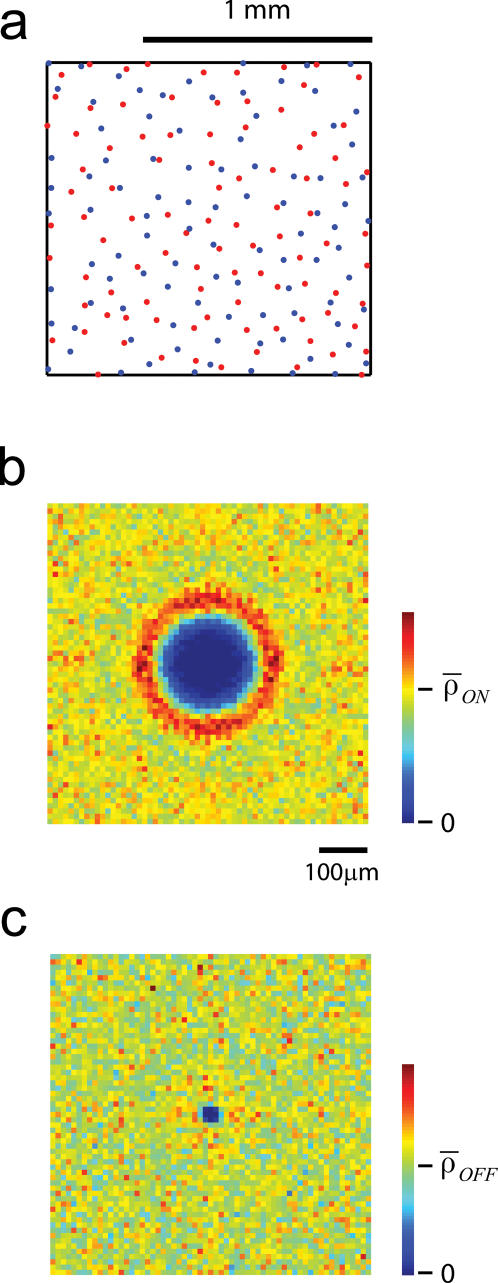
Spatial statistics of X retinal ganglion cell mosaic. (a) The result of simulating the layout of the RGC mosaic of ON-center (red) and OFF-center (blue) ganglion cells using the method of Eglen et al (2005). The simulation captures the property that ON/OFF cell pairs lying in close proximity of one another. (b) Conditional density of ON-center cells given that one ON-center cell is located at the center of the image. The shape of the conditional density illustrates that there is a disk of low density surrounding the cell, followed by a rim of high density and then decaying back to the average level. This is the behavior expected from a quasiregular distribution of cell bodies. (b) The conditional density of OFF-center cells given that one ON-center cell is located at the center of the image. The density is flat except for the fact that it dips at the center, indicating that cells bodies cannot overlap.

The possibility that the structure of the retinal ganglion cell (RGC) mosaics could influence the development of receptive fields in the cortex was first formulated by Wassle and co-workers [49]. These investigators noted that nearest neighbors on the X-cell RGC mosaic tend to be of opposite sign, generating ON/OFF pairs in close proximity (Fig 1a). As a consequence of this property, if cortical cells were to pool in space a small number of nearby receptive fields, the result would be the sum ON and OFF Gaussian receptive fields slightly displaced in space (ignoring the surrounds). This would generate a simple-cell RF with a preference for orientation. Soodak (1987) elaborated on this idea and showed that such a model generates orientation maps of periodicity comparable to those observed experimentally [29]. I recently confirmed these findings and demonstrated that a modified version of the model further explains aspects of the statistics of monosynaptic connectivity between the LGN and the cortex [30], [45], [46]. These encouraging results motivated the present study, where I attempt to take these ideas a step forward by asking if the model can account for the structure of various cortical maps and their mutual relationships.

Our discussion will center primarily on how receptive fields and cortical maps are seeded by the initially dominant input from the contralateral eye. The hope is that once the receptive fields and maps generated by the contralateral eye are well understood within the context of the theory, we can proceed to investigate how the delayed ipsilateral input, and activity-dependent processes, modify these initial structures during the critical period.

## Results

### Model description

Here I consider a simplified version of the statistical connectivity model [30] that reduces to the original proposal by Soodak [29]. The formal relationship between this simplified model and the full statistical connectivity model, as well as the selection of the parameters, are described in detail in *Experimental Procedures* below.

The first layer of the model consists of a simulation of the layout of the X retinal ganglion cell mosaic. The two key features of the spatial statistics of the mosaics the model must capture are: (*a*) the quasi-regularity of the arrays and (*b*) the functional independence between ON/OFF mosaics. Quasi-regularity implies that if we know that a cell is located in one specific location *P*, then the density of cells of the same class as a function of the distance from *P* is initially low at small distances, increases to peak above the mean density, and then settles down to the mean density at large distances (Fig 1b). The functional independence of the arrays means that knowing the location of one ON cell only tells us that an OFF cell cannot lie at the same location (Fig 1c) [51]. The simulated retinal ganglion cell mosaic in Fig 1a shows how these two features generate pairs of ON/OFF cells in close proximity.

Receptive field centers are modeled as (isotropic) Gaussians functions of appropriate size to replicate the experimentally measured coverage factor (representing the average number of receptive fields covering one point in visual space) [79]. Geniculate cells are assumed to get input from only one X-RGC [80], [81]. This is a reasonable approximation because, even if some geniculate cells receive more than one afferent, the receptive fields of their inputs overlap almost entirely [82]. Thus, the (adult) cortex can be thought as effectively sampling directly from the RGC mosaic. Afferents to the cortex are assumed to reflect the retinotopic organization of the RGC mosaic.

The second layer in this simplified version of the model represents layer 4 of the cortex. Each cortical cell weights the input afferents by a (isotropic) Gaussian function of the distance between the afferent and the cell body location. The spatial extent of the Gaussian, determined by its standard deviation 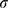, is such that only a few geniculate cells contribute strongly to the cortical receptive field. The receptive field of the cortical cell is simply the linear sum of all of its weighted afferents. There is no thresholding or spiking in the simulated cortical cells. Thus, all the results of the model are best interpreted as referring to the feed-forward intracellular response in first-order layer 4 cells.

After computing the receptive field at each cortical location, a number of its characteristics, including its center in the visual field, preferred orientation and spatial frequency were computed as described in *Experimental Methods*. Having this information at hand, I investigated the predicted relationships among the retinotopic, orientation and spatial frequency maps, as well as how tuning for orientation varies as function of location within the orientation map.

### Receptive fields and orientation maps

**Figure 2:**
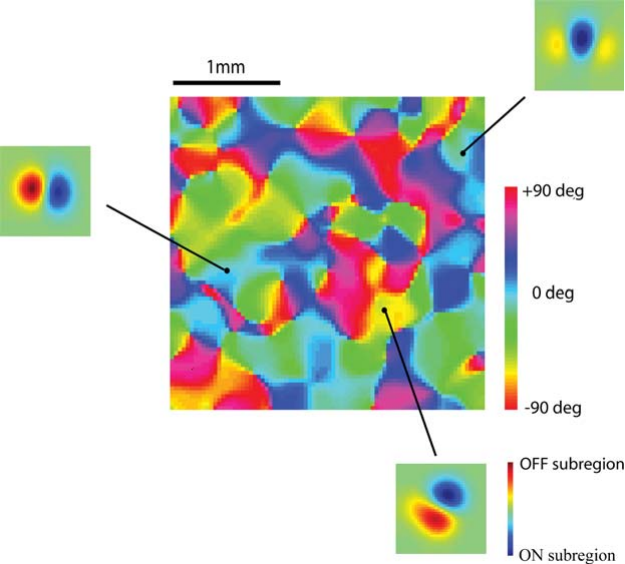
Orientation maps and simple cell receptive fields generated by the model. The orientation map and representative receptive fields at three locations on the map are shown. Receptive fields are represented in a pseudo-color map such that ON-subregions appear in red and OFF-subregions appear in blue. The horizontal scale bar represents 1mm of cortex.

To summarize some of our previous results [30], one finds that despite the fact that there are no anisotropies invoked anywhere in the model (i.e., the receptive fields of the geniculate cells and connectivity function are both circularly symmetric), the resulting receptive fields can be tuned for orientation (Fig 2). The reason is simply that the tendency for ON/OFF center cells to cluster in the RGC mosaic, the relative low coverage factor, and the pooling of a small number of afferents combine to generate oriented receptive fields.

Interestingly, because orientation selectivity arises primarily due to the pairing of ON- and OFF-center cells as nearest neighbors in the RGC mosaic, the model explains the tendency of simple cells to have two effective sub-regions [83], [84] and odd-symmetry [83], [85]. Similarly, the model predicts that blocking one of these classes of cells should prevent the development of normal orientation selectivity [86]. As explained in a previous study, the model falls short in accounting for the full elongation of the sub-regions as measured by the first-order kernels in reverse correlation experiments, but is compatible with the distribution of afferents along the a RF sub-region [30], [45]. The simulated orientation maps exhibit many of the qualitative features observed in the data, including orientation singularities (pinwheels), fractures, saddle points, and linear zones [29], [30].

### Retinotopy is linked to the density of retinal ganglion cells

**Figure 3:**
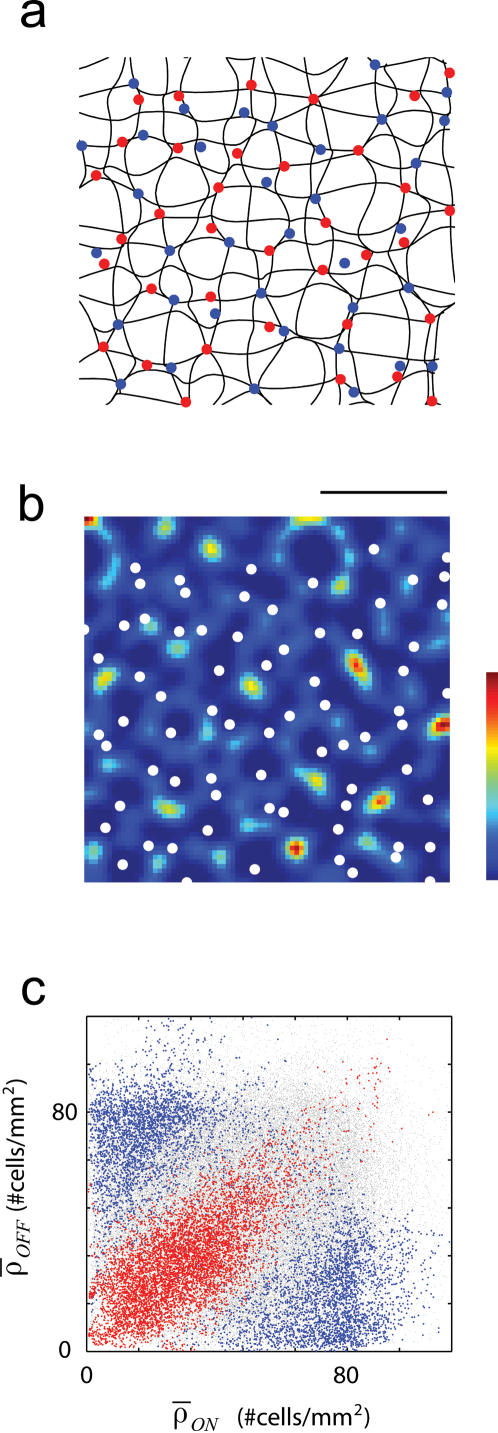
Retinotopy is linked to the local density of retinal ganglion cells. (a) Local distortions in the retinotopy are linked to the local RGC density. The diagram illustrates the location of the RGCs in one patch retina along with the locations in visual space of the centers of cortical RFs whose cell bodies lie on a perfectly square grid on the cortex. All regions bounded by adjacent vertical and horizontal contours map to the same square on the cortical grid. Thus, large regions correspond to locations of low magnification factors and small regions correspond to locations of high magnification factors. (b) The distribution of the Jacobian is such that it sprinkled with discrete regions of local maxima. These regions of rapid change in the cortical RF locations correspond to areas of low densities in the RGC mosaics (both ON and OFF cells are shown by the white dots). (c) The figure shows a scatter-plot of (*ρ_ON_, ρ_OFF_*) while indicating in red those locations with the highest 10% (in red) and the lowest 10% (in blue) values of the Jacobian.

To study the retinotopic map in the model imagine superimposing a perfectly square grid on the surface of the cortex. For each neuron on the grid we can find out the location of its receptive field center in visual space (as defined by its center-of-mass). The result of plotting the centers of receptive fields in visual space in one instance is shown in Fig 3a. Superimposed on the grid are the locations of the RGC centers (ON-center (red) and OFF-center (blue) dots). Note that regions in the visual field bounded by adjacent horizontal and vertical contours map to the same surface area in the cortex. Therefore, large regions correspond to areas of *low* local magnification factors (measured in mm^2^/deg^2^) while smaller regions represent areas of *high* magnification factors. It is apparent from the diagram that areas of low magnification are those where the density of RGC input is low, while areas of high magnification correspond to areas where the density of RGC is high. In some way, this is reminiscent of the relationship between RGC density and cortical magnification that is known to exist at a coarse scale [87]. The model suggests that a similar relationship might be expected at small spatial scales, so that fluctuations in the local RGC density should affect the local cortical magnification factor.

One way to quantify this effect is to define (*u, v*) as the coordinates on the cortex and (*x, y*) the coordinates of the visual field. The map *T*: (*u, v*)→(*x, y*) describes the location of the receptive field's center-of-mass in the visual field at any given cortical location. The area of visual space represented by small area of tissue around a cortical point is then given by the determinant of the Jacobian matrix: 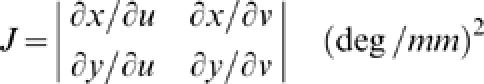



This number corresponds to the inverse of the local (area) magnification factor and is directly related to the rate of change of RF position across a unit of cortical distance [88], [89]. Interestingly, the distribution of the Jacobian is punctated by local maxima (Fig 3b). When the RGC input (represented by the white dots in the Figure) is laid on top of the Jacobian distribution it appears evident that local maxima of the Jacobian coincide with areas of low RGC density.

A better understanding of the Jacobian dependence on the local density of ON/OFF cells is gained by highlighting the locations in the (*ρ_ON_, ρ_OFF_*) plane achieving the highest and lowest values of the Jacobian (Fig 3c). In this scatter-plot, red dots indicate the locations above the 90^th^ percentile for the Jacobian distribution, blue dots represent those below the 10^th^ percentile, and gray dots correspond to intermediate values. It can be seen that the highest values of the Jacobian (lowest cortical magnification) tend to occur when both *ρ_ON_* and *ρ_OFF_* are low. The lowest values of the Jacobian (highest magnification) are attained in regions where one of the densities is high and the other low.

These results can be understood intuitively. The quasi-regularity of the arrays implies that areas where *both ρ_ON_* and *ρ_OFF_* are low will be surrounded by a rim with above average densities of cells. The receptive fields in the region of cortex receiving afferents from a retinal patch with low RGC densities will see their receptive fields dominated by inputs coming from the surrounding rim. Thus, as one moves on the cortex across a region with low RGCs densities the cortical RFs will rapidly shift their position form one side of the rim to the one diametrically opposed. This, of course, implies a large value of the Jacobian. In contrast, if at one retinal location *ρ_ON_* is high and *ρ_OFF_* is low then, by the quasi-regularity of the arrays, one expects the opposite relationship to hold in the near neighborhood. This local ‘anti-phase’ relationship between the densities allows for a relatively uniform *total* density across a retinal patch, so the cortical receptive field centers receiving input from this area shift at a low constant rate.

### Regions of rapid retinotopic change tend to align with pinwheels

**Figure 4:**
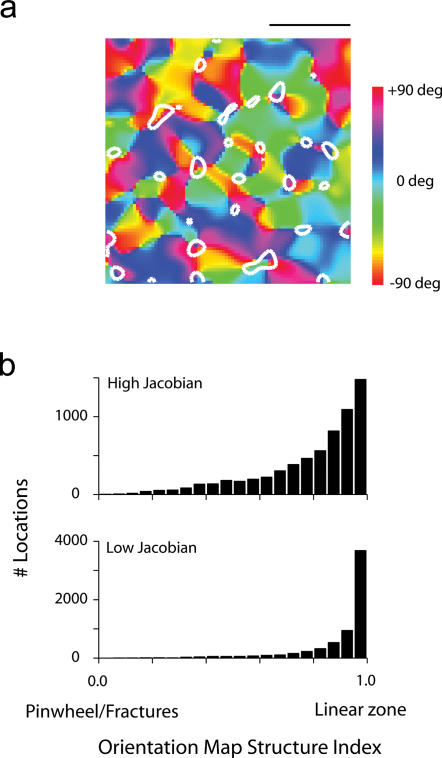
Regions of rapid retinotopic change tend to align with regions of rapid orientation change. (a) A example of an orientation map along with contours bounding the locations with the top 10% values of the Jacobian. There appears to be a tendency for these regions to fall near pinwheels or fractures. (b) Distribution of the orientation structure index (where linear zones attain a value of one, and pinwheels/fractures a value near zero), for regions with high and low Jacobian values.

Some experimental results have suggested that the rate of change of RF centers is correlated with the rate of change of their orientation preference [88]. To investigate if such effect is present in the model I analyzed where regions of rapid change (detected as the local peaks of the Jacobian) fall within the orientation map. The simulations indicate that the peaks of the Jacobian tend to align with regions of rapid change in the orientation map (either singularities or fractures). The white contours in Fig 4a bound regions above the 90^th^ percentile for the orientation selectivity index. Visually, it appears as if these regions tend to align with pinwheels. To evaluate this relationship quantitatively we defined a local index of map structure that approaches one in smooth linear zones and is close to zero near pinwheels and fractures (see *Experimental Methods*). The distribution of the orientation map indices for regions with high Jacobian values (above the 90^th^ percentile) versus low values (below 10^th^ percentile) is shown in Fig 4b. The results show that regions of rapid orientation change, near pinwheels or fractures, tend to have high Jacobian values. However, it is also evident that regions with high Jacobian values can also be found near linear zones (Fig 4b). Thus, while a trend is present in the simulations, it is not as strong as one might have expected from some experimental results [88]. It is possible that the discrepancy between the experimental findings among different laboratories on the relationship between the retinotopic and orientation maps [88]–[91] is the result of a limited sample of map locations. It is expected that two-photon imaging of retinotopy and orientation preference will likely settle the exact relationship between retinotopy and orientation maps soon.

It is worth noting that the link between retinotopy and the structure of the orientation map in the statistical connectivity model is a direct consequence of the feed-forward thalamocortical convergence and fluctuations in RGC density – an explanation that differs substantially form models that postulate intra-cortical connectivity as being the critical element for the establishment of this relationship [92].

### Orientation selectivity correlates with local map structure

**Figure 5:**
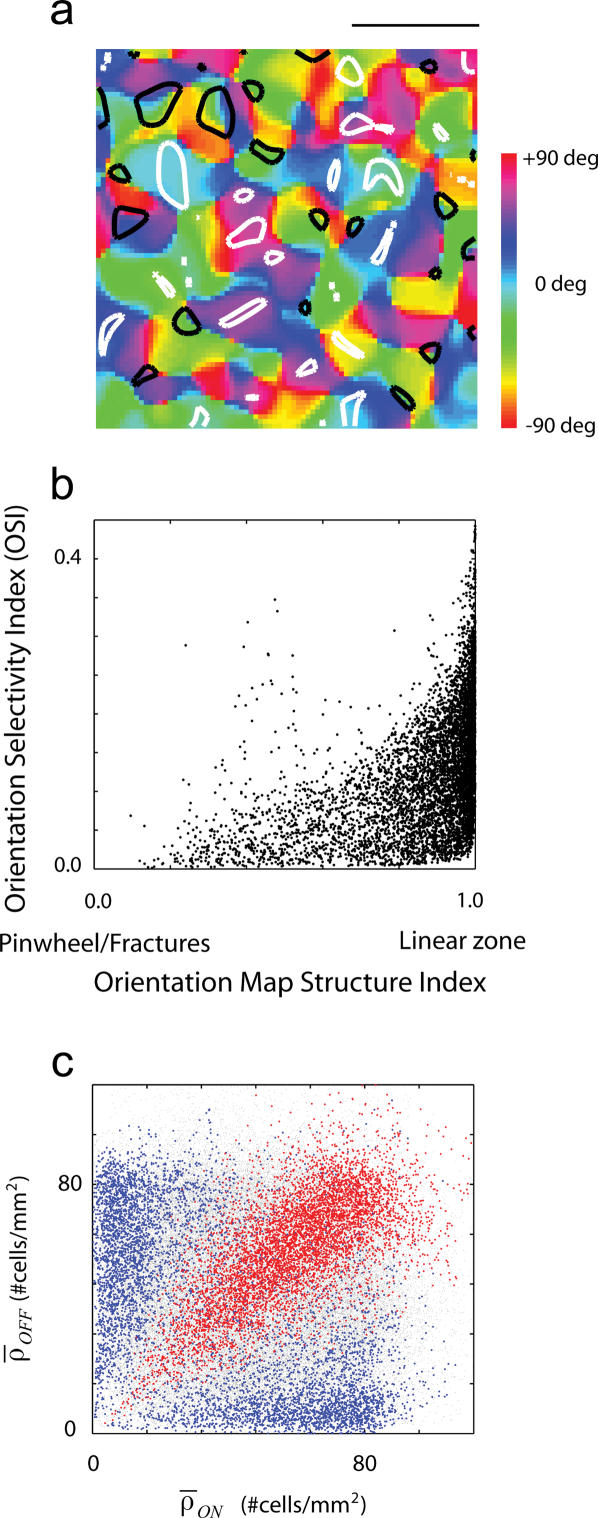
Dependence of orientation selectivity as a function of location in orientation map and retinal ganglion cell density. (a) Example of an orientation map generated by the model along with regions of high (white contours) and low (dark contours) selectivity. It is apparent that there is a tendency for the white contours to lie in linear regions, while dark contours tend ot lie close to pinwheels or fractures. (b) Scatter-plot between the map structure index and the orientation selectivity index across a simulated piece of cortex. Regions in the map with rapid orientation change tend to have low orientation selectivity. (c) Dependence of orientation selectivity as a function of the local density of ON-center and OFF-center cells. Well tuned cells lie in regions where the densities are both high, as these are regions that will tend to generate RF with two or more subregions. Locations where only one class of cell dominates will tend to generate RFs with one dominant subregion and be broadly tuned for orientation.

A natural question to ask is if there is any relationship between the selectivity of neurons for orientation and their location in the orientation map [93]–[96]. Given a simulated receptive field, an orientation selectivity index (OSI) was defined such that well-tuned cells have an OSI near one, while untuned cells will have an OSI near zero (*Materials and Methods*). Fig 5a shows an orientation map from one simulation along with the isocontour levels corresponding to the 10th (black contours) and 90th percentile (white contours) of the orientation selectivity index. The regions enclosed by the white contours correspond to regions of high orientation selectivity, while the dark contours enclose areas of low selectivity. There is a clear trend for regions of high selectivity to reside within linear zones of the map, while cells with low selectivity are present near pinwheels or fractures. To better quantify this relationship, I plotted the selectivity of individual cells as a function of the map structure index [93]–[95]. There is a correlation between these two variables indicating that well-tuned cells tend to be located within the linear zones, while broadly tuned cells tend to be located near the pinwheels.

Once again, it is worth emphasizing that the dependence of orientation selectivity with the local map structure is solely the consequence of the feed-forward architecture of the model. This finding is important because it indicates that variations of tuning selectivity across the orientation map do *not* necessarily have to arise as a consequence of differences in the local feedback signal within the cortex, which has been a key assumption in interpreting some experimental data [93], [95], [96].

### Orientation selectivity correlates with local RGC density

Intuitively, orientation selectivity is expected to be higher in receptive fields that pools ON and OFF receptive fields in close proximity. This arrangement is more likely to occur when the local densities of both ON and OFF cells are high. Accordingly, the mean orientation selectivity as a function of the local RGC densities shows an increase along the diagonal, as both *ρ_ON_* and *ρ_OFF_* increase (Fig 5c). High density of any one class of ganglion cells by itself is not sufficient to generate high orientation selectivity, as these inputs will tend to generate RFs with only one dominant sub-region.

### Spatial frequency maps and their relation to orientation selectivity

**Figure 6:**
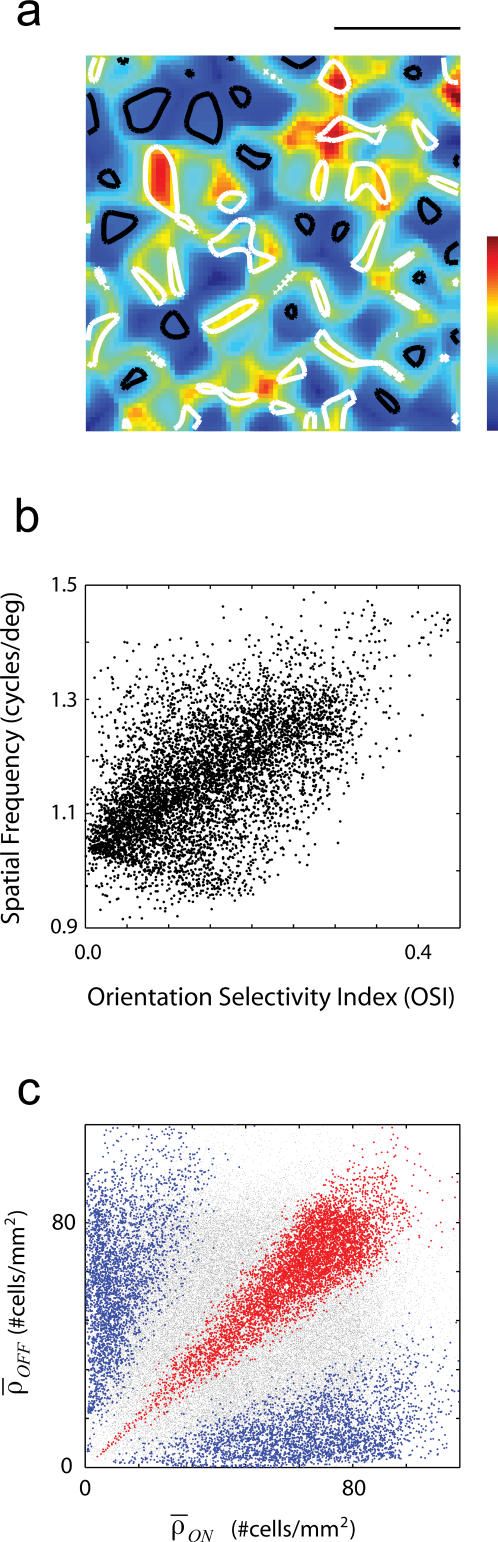
Spatial frequency maps and their relation to selectivity for orientation. (a) Spatial frequency map with regions of high (white contours) and low (dark contours) orientation selectivity superimposed. Regions that are well tuned for orientation tend to have higher spatial frequency preference. (b) Scatter-plot showing a positive correlation of orientation selectivity and spatial frequency preference. (c) Dependence of spatial frequency preference as a function of the local density of ON-center and OFF-center cells.

The model further predicts the existence of spatial frequency maps of periodicity comparable to that of the orientation map (Fig 6a) [10], [34]. An examination of the RFs generated by the model reveals that the frequency maps are determined mostly by changes of the tuning curves at low spatial frequencies, as the high frequency cut-off is effectively determined by the center size of the geniculate afferents. Receptive fields that have side-by-side subregions of opposite sign are band-pass in spatial-frequency, while receptive fields that have mostly one dominant subregion are low-pass. The center-of-mass of the spatial-frequency tuning curve (which is used to define the preferred spatial frequency of the RF) shifts to higher spatial frequencies as the response to low spatial frequencies is suppressed. This mechanism generates a correlation between the spatial frequency map and selectivity for orientation, as observed experimentally [83], [84], [97].

There is a positive correlation between spatial frequency preference and orientation selectivity (Fig 6a). The figure shows an example of a spatial frequency map in one instance of the model on which areas of high (white contours) and low (dark contours) orientation selectivity are superimposed. It is apparent that cells that are well-tuned for orientation tend also to have a higher spatial frequency preference. This is most clearly shown in the pixel-by-pixel scatter plot of orientation selectivity versus peak spatial frequency (Fig 6b). Finally, as both orientation and spatial frequency depend on the effective number of subregions of the receptive field, the spatial frequency preference increases along the diagonal in the (*ρ_ON_, ρ_OFF_*) plane (Fig 6c). Regions where one of the densities is high and the other low generate RFs with only one dominant subregion and tend to be low-pass in spatial frequency (blue areas in Fig 6c).

### Pinwheels align with extreme spatial frequency preferences, but the reverse is not true

**Figure 7:**
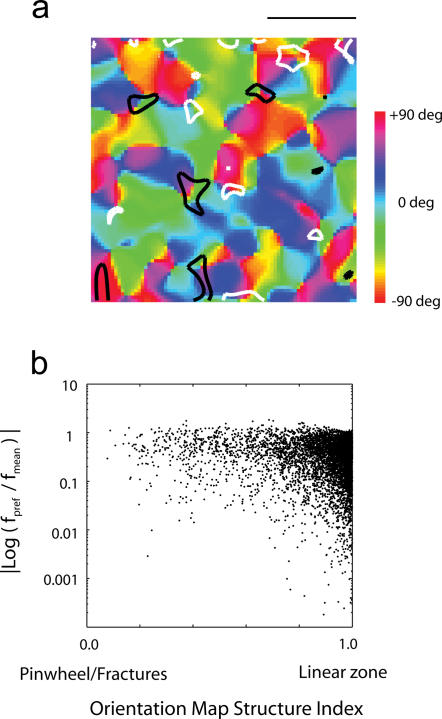
Relationship between orientation and spatial frequency map. (a) Example of an orientation map along with areas of high (white contours) and low (dark contours) spatial frequency selectivity. There appears to be a tendency for both high and low spatial frequency domains to align with pinwheels. (b). Plotting the absolute deviation of the local spatial frequency from the mean across the population as a function the local map structure confirms that regions with low map structure indices, corresponding to pinwheels/fractures, tend to be associated with an extreme (either low/high) spatial frequency location (the opposite is not always true).

Experimental results have suggested a tendency for regions of high and low spatial frequencies to overlap with the pinwheels in the orientation map [10]. This predisposition is also present in the maps generated by the model (Fig 7a), where dark (low frequency) and white (high frequency) regions tend to be align with regions of rapid orientation change. This may be better appreciated by plotting the index of local map structure versus the absolute deviation of preferred spatial frequency from the mean preferred frequency across the entire population (Fig 7b). Here, one observes that regions with low map structure indices (corresponding to pinwheels and fractures) tend to have large deviations in spatial frequency, implying the presence of RFs with either low or high spatial frequency preference in these regions. Note that the reverse is not necessarily true: extreme low/high spatial frequency domains do not necessarily have to overlap with regions of rapid orientation change.

### Cytochrome oxidase blobs

Another prominent feature of the anatomical organization of primary visual cortex are the cytochrome oxidase (CO) blobs [98]. In cats, cytochrome-oxidase blobs develop around 2 weeks of age without the need for visual experience [99], [100]. There is a tendency for CO blob centers to be associated with areas of reduced orientation selectivity, low spatial frequencies and monocular regions [33], [34], [99], [100]. The hypothesis that cytochrome-oxidase (CO) expression in the cortex could be caused by the clustering of cells with broad selectivity has been considered in the past [98]–[101]. However, because CO patterns are present in visually inexperienced animals, this proposal was regarded unlikely [98]. Yet, it remains possible that these structures could arise supported by spontaneous activity present in the retina and the LGN during development. I asked if regions of high metabolic activity may result from a clustering of cells with low selectivity. To investigate this question I first devised a measure of ‘metabolic activity’ defined by the average activity of the neurons when exposed to ensembles of natural stimuli (see *Materials and Methods*).

**Figure 8:**
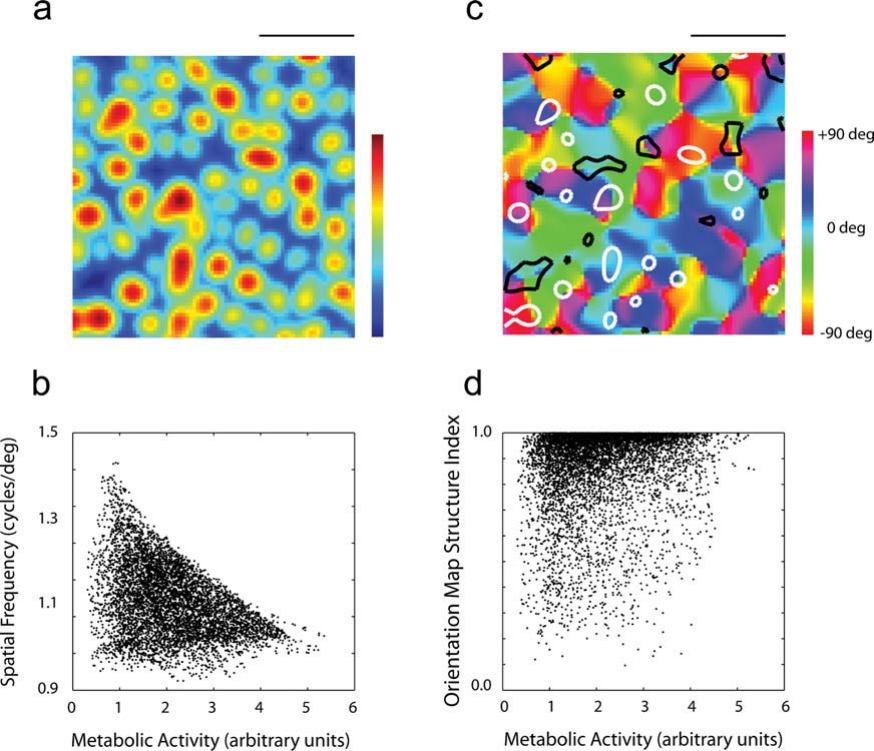
Cytochrome oxidase blobs. (a) The distribution of a metabolic activity index from the RFs generated by the model shows a clustering of cells with broad selectivity generating a blob-like pattern. (b) Regions of high metabolic activity (putative location of the CO blobs), as expected, tend to align with regions of low spatial frequency preference. (c) An example of an orientation map along with regions of high (white contours) and low (dark contours) metabolic activity. There appears to be no obvious relationship between the two. (d) A closer examination by plotting the metabolic activity index versus the orientation map structure index shows that regions of rapid orientation change (pinwheels/fractures) tend to align with regions of low metabolic activity, consistent with the experimental finding of Shoham et al (1997).

The model yields a distribution of metabolic activity across the cortical surface having a characteristic blob-like pattern (Fig 8a) reminiscent of CO blobs [12]. Not surprisingly, given the definition of the metabolic activity index, one finds higher metabolic activity in areas of low-spatial frequency tuning (Fig 8b). A natural question is where these regions lie within the orientation map. Surprisingly, the peaks of metabolic activity (the putative location of the CO blobs) do *not* appear to align with pinwheels in the orientation map (Fig 8c). Instead, one observes a tendency for pinwheels to align with regions of *low* metabolic activity (but the reverse is not always true – low metabolic activity does not always imply the presence of a pinwheel). This is confirmed by a pixel-by-pixel scatter-plot of these two variables (Fig 8d). Remarkably, this is the same trend found experimentally: it has been reported that most pinwheel centers (83%) are found in inter-blob regions [12].

## Discussion

The possibility that statistical connectivity seeds the initial architecture of primary visual cortex has been introduced and discussed. During the critical period, activity dependent mechanisms are expected to maintain and modify these initial structures. An important question is to what extent the initial condition determines the final structure of the cortex during normal (and abnormal) development. In principle, it is possible that self-organization could completely wipe out any trace of the initial condition (which, of course, would render the present proposal meaningless). This seems unlikely, as no dramatic changes are observed in the in the evolution of the maps during normal development. Instead, our findings suggest that the initial condition (instantiated via statistical connectivity) has a substantial influence on the final structure of receptive fields and cortical maps.

### Extension of the model to binocular input

The model in its present form suggests a way in which the early input from the contralateral eye could set up a blueprint for the cortical architecture. More work remains to be done, as a full description of the developmental process demands an explanation for how the input from the ipsilateral eye ‘goes along’ with this blueprint. Three key questions in search for an answer are: (*a*) What generates the pattern of ocular dominance before the onset of the critical period [55]? (*b*) How can the ipsilateral eye gain any cortical territory if initially the contralateral eye dominates? (*c*) How are the orientation preferences for both eyes matched [36]?

Now that we have gained some insight into the structures generated by the contralateral input I have begun to explore a new hypothesis that may provide answers to some of these questions. I suggest that ocular-dominance columns may reflect the fluctuations of the RGC densities in corresponding retinal locations of the two eyes. In locations where the RGC density is high for the contralateral eye but low for the ipsilateral eye one may expect contralateral dominance. However, in regions where the density of the ipsilateral eye is higher than that of the contralateral eye, it may be possible for the ipsilateral eye to take over cortical territory. Binocular areas would be expected in regions where the RGC densities are approximately equal. Note that one extreme case where this clearly occurs is in the optic disk representation. It is also known that such mechanism can work at very fine spatial scales, as demonstrated by the cortical representation of ‘angioscotomas’ caused by cast shadows of retinal blood vessels [102], [103].

**Figure 9:**
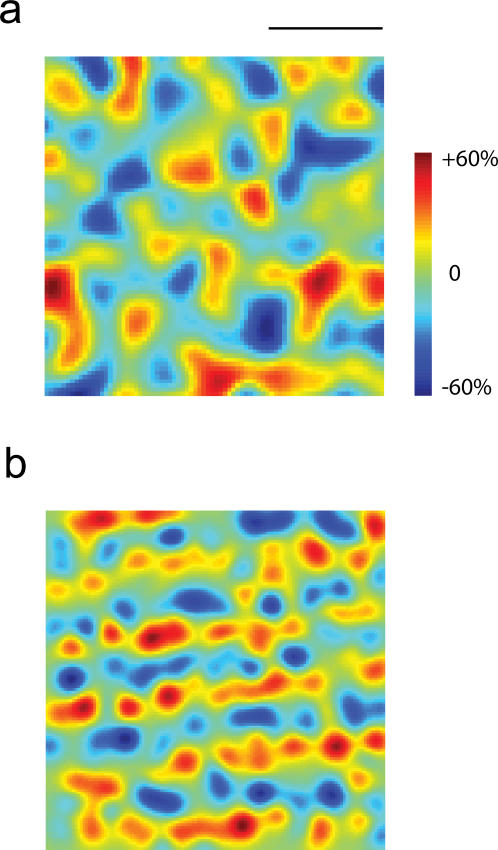
Relative RGC density as the seed for ocular dominance columns. (a) The relative density of left/right eyes at a scale relevant for the formation of receptive fields shows large fluctuations, in the order of 60%, which could seed the formation of ocular dominance columns despite an early contralateral dominance. (b) When anisotropy in the retinotopy map is simulated, the result is a relative density having a banding pattern similar to that observed in some primates.

To start evaluating the merits of this idea I computed the relative density of the ipsilateral versus contralateral eyes, defined as *OD* = (*ρ_ipsi_*−*ρ_contra_*)/(*ρ_ipsi_*+*ρ_contra_*); where *ρ = ρ_ON_*+*ρ_OFF_*. The relative density has blob-like pattern with similar periodicity to the orientation and spatial frequency maps (Fig 9a). The relative change in the density favors one or the other eye by as much as 60%. Therefore, there are significantly large fluctuations in the local density of the two eye inputs that could potentially create a blueprint for the establishment of ocular dominance columns. If the local magnification factor is anisotropic (Fig 9b, shows a case of 2∶1 anisotropy) the model predicts a stripe pattern of ocular dominance such that the orientation of the stripes is orthogonal to the direction of higher magnification, as generally seen in the primate [21], [104].

This hypothesis fits the available data in another interesting way. Recall that the model predicts that regions of low RGC densities imply a large Jacobian, and that pinwheels tend to align with regions of high Jacobian values (Fig 4). Thus, if regions of low RGC contralateral eye densities are preferentially taken over by the ipsilateral eye, as proposed, some of the peaks of ipsilateral eye dominance will likely fall within pinwheel centers. Remarkably, this is exactly what the experimental data show [105].

If the hypothesis about the origin of ocular dominance columns is correct, then ocular dominance peaks are locations where RGC densities for the contra and ipsilateral differ the most, and given that we know that RGC densities determine the local magnification factor (Fig 3), then it must be also the case that at the peak of ocular dominance columns the magnification factors for the two eyes differ the most. Obtaining precise retinotopic maps with cellular resolution at various locations within the ocular dominance map could be used to test this prediction.

Another important question is how the orientation preference for the two eyes and the RFs of binocular simple cells are matched [36], [106]. Following previous work on the topic, one may postulate that spontaneous activity, along with correlation-based mechanisms, are responsible for this process [107]. Thus, activity dependent mechanisms could work to wire those inputs that match the (already laid out) contralateral RF driven by between-eye correlations in spontaneous activity of geniculate neurons [108].

### Functional maps in other species

While developing the model in the cat makes sense, one cannot avoid to ask some questions that relate to the model's ability to explain the structure of maps in other species [26]. How could a salt-and-pepper pattern co-exist with orientation selective cells in the statistical connectivity model [25], [109]? One could speculate that in species lacking orientation maps coverage ratios of ON/OFF lattices is higher (there is more overlap) but that the probability of connectivity is lower. If the average number of inputs were about two, then about half the time a cell would pool from one ON and one OFF-center input, generating RFs similar to those displayed in Fig 2. However, as each cell is likely to pool a different pair of inputs, the result would be a set orientation neurons within a salt-and-pepper map.

Why are maps so variable across individuals of a single species? Why, on some individuals, are maps present in some areas of the visual field and not others [26]? Here, one could conjecture that it is the variability in the RGC densities, coverage factors, and spatial pooling across individuals and even across the visual field of the single individual, could account for such variability. Assessing these ideas would require a careful extension of the statistical connectivity model to other species based on available data.

### Relationship to previous work

There has been extensive theoretical work aimed at studying the properties of cortical maps and their development. A detailed discussion of these models is outside the scope of this paper (see Swindale (1996) for an extensive review of the topic). Here I limit myself to a brief description of the classes of models that have been considered in the past, and how they differ from the statistical connectivity approach.

First, there is a class of correlation-based models [58], [59]. These models can account for a large number of experimental findings about how manipulations of early sensory experience influence the final structure of the maps [42], [56], [59], [60], [66], [67], [107], [110]–[120].

The role of correlation-based mechanisms in establishing the initial blueprint of the columnar architecture and receptive fields in the young cortex is, however, not well established. The presence of within and between-eye correlations at small spatial scales is critical for this mechanism to work [59], [107]. In the ferret, it has been shown that while between-eye correlations are present, they are sign-independent. In other words, there is no difference between ON/ON and OFF/OFF correlations to those of ON/OFF pairs [108]. Recent measurements further demonstrate a simple fall-off of correlations with distance in the developing LGN [121]. This is contrary to the Mexican-hat profiles predicted by some activity-dependent models. These findings call for nontrivial constraints to be incorporated into learning rules, such as split constraints for ON- and OFF-center afferents, to allow learning models to develop simple-cell receptive fields [121].

The experimental finding that both ON and OFF inputs into the cortex are required for the development of orientation columns [86] has been considered strong evidence for a correlation-based origin of the orientation map [59]. However, as we explained above, statistical connectivity provides a simple alternative explanation. Two additional challenges for the involvement of correlation-based mechanisms in setting up the blueprint for the orientation and ocular-dominance maps are: (*a*) simple cells with segregated ON/OFF receptive fields emerge from the *very beginning* without going through a stage of significant ON/OFF overlap [53]; (*b*) ocular dominance columns develop from an initial state where the cortex is strongly dominated by contralateral input [36], and it is difficult to envision how Hebbian-based models could easily overcome this strong bias. Statistical connectivity provides an answer to the first question, and I have offered a novel hypothesis regarding the emergence of ocular dominance columns that may address the second one.

Second, there is class of dimension-reduction models that tend to be more abstract that the mechanistic explanation offered by statistical connectivity. These models begin by defining an input space, with some coordinates being linear (representing position or spatial frequency) and others circular (representing orientation), need to be mapped to the two-dimensional plane in some optimal fashion [61], [62], [64], [116], [122]–[126]. Mappings that ensure measures of *continuity* and *completeness* can be searched by a number of different algorithms, such as self-organizing map or the elastic-net algorithm. Continuity refers to the condition where nearby cells have similar properties. Completeness ensures that all combinations of the features represented on the map are distributed uniformly over visual space. It is interesting that these two simple principles can account for a number of features in the cortical maps. Furthermore, experimental measurements support the idea that the maps are organized in such a way that they optimize a quantitative measure of completeness, called the *coverage uniformity* [125], [127]–[129]. A main difference with these methods is that statistical connectivity postulates a wiring mechanism that yields the feature maps and their relationships automatically; no assumption about the existence of a set of feature maps is needed.

Third, there is work that relies on the minimization of overall wiring length between cells as the main drive behind map formation [122], [130]–[132]. These studies show that a desired distribution of connectivity among cells with different orientations determines the features of the orientation map. For example, the requirement of a uniform connectivity results in a ‘salt and pepper’ map as seen in rats, while a mixture of a Gaussian and a uniform distribution yields maps with the characteristic pinwheel structure seen in other species. The concept is appealing due to its simplicity and because it predicts that functional connectivity, as estimated by electrophysiological methods, should correlate with map structure. In contrast, the origin and relationships between maps in the statistical connectivity model is independent of the cortical interconnections. Note, however that one assumes that intracortical wiring length is dominated by local connections, then statistical connectivity would predict a mixture of a Mexican hat and a uniform distribution between orientations as well.

Finally, it is worth highlighting a recent theoretical model of a feed-forward architecture where, under some mild symmetry assumptions, the authors show that there must be a necessary link between orientation and retinotopy [133]. The model is limited in that it only considered cells of one type (either ON or OFF). Nevertheless, this interesting work could potentially be extended to deal with both ON/OFF cells and be applicable to the analysis of the statistical connectivity model.

### Testing the model

Statistical connectivity accounts for a number of published trends in the relationships between the cortical maps. Yet, these findings could be regarded as ‘circumstantial evidence’ it is important to ask what experiments could provide the strongest possible support for the theory. As discussed above, a central role in the model is played by the local densities of the X retinal ganglion cells, which strongly influence the local retinotopy (via the Jacobian), the selectivity of the receptive fields, and the structure and relationship between the various maps. An ideal experiment would be one where the retinotopic and orientation maps are measured with single cell resolution along with a reconstruction of the X retinal ganglion cell mosaic from the contra-lateral eye in a region representing the same location of visual space. Given that the structure of receptive fields and maps may be subsequently modified via the ipsilateral input, these experiments are best interpreted if the ipsilateral eye is enucleated early and the cortex responds exclusively to contralateral input (Farley et al, *Soc for Neuroscience Meeting*, Abstract #545.2, 2006). Findings demonstrating a dependence of cortical structures with RGC density as predicted by the model would constitute a convincing piece of evidence in support of statistical connectivity. Of course, with a full reconstruction of the mosaics, it should be also possible to predict the exact structure of the orientation and retinotopic map. These experiments are not trivial, but are not outside the realm of present techniques. Similarly, the reconstruction of the X RGC mosaics of both eyes in corresponding retinal regions could be used to test the hypothesis that fluctuations in RGC densities of the two eyes correlates with the pattern of ocular dominance columns.

### Limitations and assumptions of the model

The model makes some important assumptions that are worth discussing. The most important, and pre-requisite for the model to work, is that there should be a well-established retinotopic map as the geniculate axons leave the sub-plate to invade the cortex. This is required for the model to explain how simple-cell RFs arise without a prior stage of significant ON/OFF overlap. Indeed, there is some experimental data supporting the notion of a sharpening of the topographic organization of geniculocortical projections before axonal incursion into the cortex [134]. It is also known that when subplate neurons are ablated, neither orientation tuning nor orientation/ocular dominance columns form in the visual cortex [135]. Thus, it is likely that a key role of the subplate is in aiding the formation of a fine retinotopic map *in advance* of geniculate afferents invading the cortical plate.

All the maps obtained in the model are derived exclusively from inputs from the X-RGC mosaic, even though it is known that some simple-cells in layer 4 also receive input from Y cells [136]–[139]. The assumption that the initial cortical structures will be dominated by X-cell inputs is justified as it has been reported that X-cells mature before Y-cells in the geniculate [140]–[142]. One would conjecture that Y-cells would then ‘go along for the ride’ to fit within the established architecture, in a similar way that the ipsilateral input is hypothesized to do.

The model assumes a uniform set of adult-like LGN RFs and ignores the fact that during development one observes a set of heterogeneous, and sometimes orientation-tuned, receptive fields [143]. This heterogeneity results from the pooling of a number of RGC afferents by single geniculate cells, while in the mature LGN only one input dominates. Tavazoie and Reid (2000) proposed that such transient developmental state of the LGN may assist in establishing the scaffolding for orientation selectivity in the cortex [143]. Their ideas, in conjunction with the ones incorporated in the present study, could potentially be merged to develop a model that accounted for both the development of LGN and cortical RFs.

Ignored was also the fact that RF centers are not perfectly isotropic and that they tend to be slightly elongated in a radial axis [144]–[147]. This effect appears to underlie a link between retinotopy and orientation bias in monkeys and humans [148]. Statistical connectivity could provide a way to gauge the degree to which the anisotropies in the RF centers of ganglion cells could bias the resulting orientation map.

The model further ignores global boundary effects that may have an influence on the map [58], [59], [104], [131], [149]–[151], but it seems doubtful that the entire map structure, away from the boundaries between cortical areas, is determined purely by such boundary conditions.

The model is ‘static’ and does not incorporate the full spatio-temporal structure of receptive fields. This limitation of the model will prevent us from looking at the development of direction selectivity maps [152]. However, it was felt there is already a wealth of data on maps other than direction of motion that could be used to disprove the model. If it turns out after these studies that the model is successful at explaining most of the data, it would certainly be worth extending the model by considering spatio-temporal receptive fields.

Finally, the model does not incorporate intra-cortical connections, which can influence the tuning selectivity of neurons within the orientation map and even on the structure of the maps themselves [93], [96], [153]–[155]. While these features could be incorporated at a later stage, our immediate goal is to understand how much of the data can be explained by a simple feed-forward convergence model before invoking additional processes.

In ignoring many of these details the objective was the keep the model as simple as possible while retaining its predictive and explanatory power. To do so, we have also restricted ourselves to a purely linear model that does not take into account LGN saturation or spiking in cortical neurons. It is unlikely that adding spiking to the model would substantially alter any of the basic properties discussed above, such as the preferred orientations or spatial frequencies and their basic relationships. However, thresholding could certainly influence the selectivity of neurons [93], [156]. Thus strictest interpretation of the model's predictions should then be in terms of the pattern of sub-threshold activity in layer 4 cells.

### Summary and implications

Statistical connectivity postulates that the spatial statistics of the retinal ganglion cells together with a simple feed-forward connectivity scheme between the thalamus and the cortex *seeds* the structure of the early receptive fields and maps in primary visual cortex. This initial state is likely to be maintained and refined during the critical period. Nevertheless, it is suggested that the adult structure should normally reflect a great deal of the initial organization.

It is appealing that such a simple model accounts for a rather complex set of data. Specifically, summarizing the result form this and previous studies [29], [30], one finds that the theory is capable of explaining/predicting: (*a*) how simple cells in layer 4 can emerge from the very beginning without going through a phase of extensive ON/OFF overlap [53]; (b) how blocking ON-center RGC cells precludes the development of orientation tuning [86]; (*c*) how geniculate cells of different signs avoid connecting to the same subregion (the ‘sign rule’) as a result of a clustering of ON/OFF afferents (Jin et al, *Society for Neuroscience Meeting*, Abstract #436.12, 2006); (*d*) how synaptic strength depends on receptive field sign and overlap; (*e*) the relative size of simple-cell subregions versus the input geniculate centers [45]; (*f*) that the majority of simple cells tend to have two effective subregions [83], [84] and a tendency for odd-symmetry [83], [85]; (*g*) the emergence of orientation columns; (*h*) the qualitative shape of the orientation maps, including the presence of pinwheel singularities and fractures; (*i*) how retinotopy could be linked to the fluctuations in the RGC density; (*j*) how the tuning for orientation depends on the location of cells within the orientation [93]; (*k*) the existence of spatial frequency maps and the fact that regions of high/low spatial frequencies align with pinwheel centers [10], [33]; (*l*) the correlation between orientation and spatial frequency selectivity in individual neurons [83], [97]; and (*m*) the existence of clustered regions of broad selectivity that could be related to the pattern of CO-blobs, and how pinwheels would tend to lie preferentially within inter-blob regions [12].

The model's simplicity, the fact that it provides a straightforward mechanistic interpretation in terms of the underlying circuitry, and the scope of the findings both explained and predicted, suggests we should consider this idea seriously as a working hypothesis for the origin of the cortical architecture. In the best case scenario, if the theory stands the test of time, we might have actually arrived at a potential explanation for the blueprint of the cortical architecture; a feat that has evaded our scrutiny for more than half a century.

## Materials and Methods

### The model

The full statistical connectivity model consists of three layers, representing the retina, the LGN and the cortex. To simulate the layout of X-RGC mosaics in the retina we have adopted the pair-wise interaction point process model (PIPP) [51]. Briefly, the method starts by randomly positioning *n_ON_* and *n_OFF_* cells on a simulated patch of retina of size *L*×*L*. Denote by *x^i^_ON_* the position of the *i*-th ON-center cell, and a similar notation for the OFF-center cells. The main body of the algorithm consists of the following loop.

#### Loop

For each ON-center cell a new candidate position is generated at random. Suppose we are considering the *i*-th ON-center cell. The new position is accepted with probability
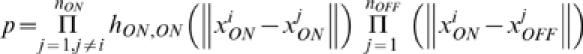



After looping over all the ON-center cells the process is repeated for the OFF-center cells, where this time the new position is accepted with probability
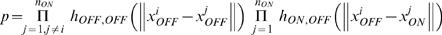



Each loop of the algorithm consists of moving all cells once. After repeating this loop about 100 times the cell positions converge to a stable pattern and the algorithm is stopped. The so-called interaction functions *h_ON,ON_*, and *h_OFF,OFF_* are defined by the parameterized function
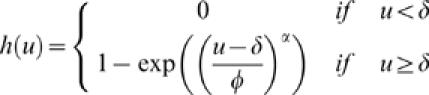

*h_ON,OFF_* was defined by the simply inhibition function,
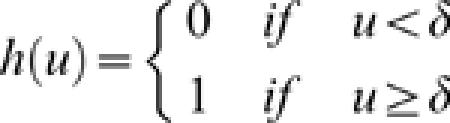



In our simulations we used δ = 20 μm, *Φ* = 90 μm, and α = 6 for both *h_ON,OFF_*, and *h_OFF,OFF_*. Details of the algorithm are presented in Engle et al (2005). These parameters were selected to match the density of cells at 6 mm (30 deg) eccentricity in the cat which is about 75 cells/mm^2^ [157]. It is worth emphasizing, however, that the findings are not dependent on an extremely accurate simulation of the RGC mosaic. Simpler methods, such as the perturbation of the vertices of two independent hexagonal lattices [30] yield similar results.

The size of the RF centers were selected to achieve a coverage factor of 3 (surrounds were ignored). The average dendritic field diameter of X ganglion cells at 6 mm eccentricity is *d* = 225 µm, corresponding to an area, *A* = 0.04 mm^2^. The standard deviation of the RF center size was determined by assuming that 4×*σ_ctr_* = *d* = 225 µm.

In the second layer of the model there are 2.5 times more geniculate cells than ganglion cells, but each receives only one input from a RGC. The receptive field of the LGN cell is assumed to be identical to that of its afferent geniculate neuron.

The third layer represents layer the cortex. Consider an arbitrary cortical location on the cortex that, for convenience, we define as the origin, (*x, y*) = (0, 0). We are given a set of geniculate receptive fields represented by *LGN_i_*. Then, a single realization of a receptive field at the cortical location is generated by 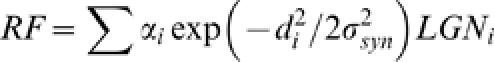. Here, *d_i_* represents the distance between the i-*th* LGN afferent and the origin, and *α_i_* are independent Bernoulli random variables such that the probability of success 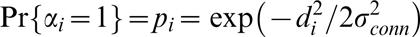. This is the full connectivity model as studied previously in Ringach (2004).

#### Model simplification

The simulations can be simplified considerably if one computes the mean receptive field at each location. The mean receptive field is the average receptive field expected over many realizations and it is given by:
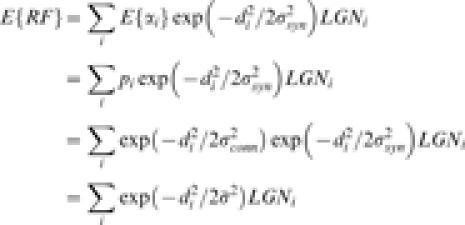



Here,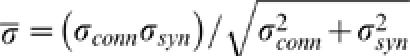. This simple relationship provides a very effective way to calculate the mean receptive fields at any cortical location that arise from statistical connectivity without the need of generating large number of RFs at each cortical site. All that is required is the calculation of a weighted sum of the geniculate receptive fields. This simplified model is identical to the one considered by Soodak (1987). Thus, the Soodak (1987) model is formally the calculation of the mean RFs in the full statistical connectivity model of Ringach (2004).

Of course, one must be cautious as the properties of the mean receptive field *do not* necessarily have to reflect the mean of the properties of the individual cells within a column. In our simulations we have carefully verified that the resulting maps and their relationships for the full and simplified models are nearly identical for the parameters selected in a number of instances.

#### Parameter selection

The selection of the parameters was done based on the fact that he lateral spread of geniculate X-cell afferents into the cortex is about 0.5 mm [136], [137], [158]. This, together with the data of Alonso et al (2001) on the decay of both the probability of connection and synaptic strength with distance (their Fig 4 and 5), guided our selection of *σ_conn_* and *σ_syn_* to be ≈0.2 mm, implying that the Gaussian function falls to 4% of its peak at 0.5 mm. To obtain periodicities of 0.8 mm in the orientation map [32] a magnification factor of 0.6 deg/mm is required in the model. This is within a factor of two of the data presented in Tusa et al (1978) which suggest a value of 0.31 mm/deg. Given the variability in the periodicity of maps and retinotopy across individuals it seems premature to conclude that this mismatch in spatial scale is sufficient to rule out the model. As discussed above, measurements of the magnification factors, orientation maps, and RGC densities in the *same* individuals are needed to test the model carefully. It is worth noting that the basic results of the model, however, are not extremely sensitive to the parameter settings: changes of the order of ±25% generated very similar results.

### Summary of receptive field properties

Given the mean RF at one cortical location, we summarize a number of its characteristics: (*a*) its center-of-mass in visual space coordinates; (*b*) its preferred orientation; (*c*) its orientation selectivity; (*d*) its spatial frequency preference; and (*e*) a measure of its average activity expected when the receptive field is stimulated with natural images, which we term the ‘metabolic activity’ index. We describe how we compute each one in turn next.

#### Center-of-mass

To compute the center of the receptive field we simply compute the center-of-mass 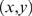 of the absolute value of the RF in the visual field coordinates. Thus,
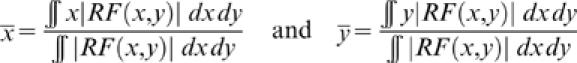



#### Preferred orientation and spatial frequency

This, and the other features below, are computed based on the Fourier spectrum of the receptive field, *RF(ω_x_,ω_y_)*. Computing the center-of-mass of the amplitude spectrum yields the resultant:
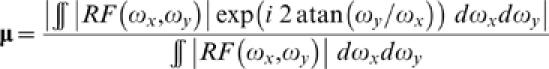



The preferred orientation is defined by half the angle of the resultant, 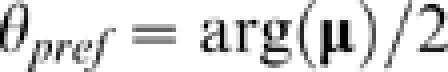, while the preferred spatial frequency is defined as the amplitude of the resultant. 
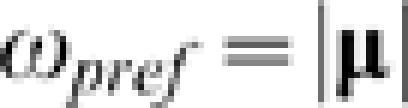
. Note that the preferred spatial frequency is *not* the peak of the spatial frequency tuning curve but rather its center-of-mass.

#### Orientation selectivity

Orientation selectivity is defined as the orientation selectivity index applied to the amplitude spectrum evaluated at the preferred spatial frequency. In other words, it is the OSI of 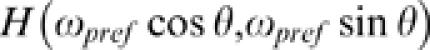, which is given by
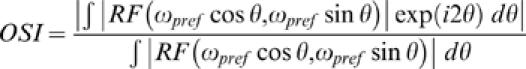



#### Metabolic activity index

The power spectrum of natural scenes falls like 1/ω^2^ [159]. The expected power of a linear receptive field in response to such image ensemble could be used to define a measure of the overall activity, or metabolic requirement. We define, 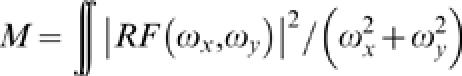, as our metabolic activity index.

#### Map structure index

We characterize the local structure of the orientation map by computing an index based on the distribution of orientation preferences in a small neighborhood of each cortical point. The index is the same as the orientation selectivity index that is used to calculate the selectivity of neurons to orientation. Given the map of preferred orientations, θ (*x, y*), and a cortical location (*x*_0_, *y*_0_), define
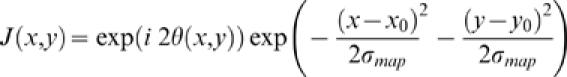



Then the index of local map structure at (*x*_0_, *y*_0_) is given by
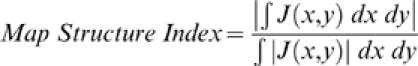



Indices close to one imply a linear zone; indices close to zero imply a region of fast orientation change – either a pinwheel or fracture. In our simulations we used *σ_map_* = 75 μm.

We have tried various alternatives to these measures. For example, one strategy we have implemented is fitting a two-dimensional Gabor function to the mean receptive field. The properties of the RF are then summarized by the fitted parameters. This procedure takes long processing times, as a nonlinear optimization problem has to be solved at each cortical location. Furthermore, in many cases (such as when the RFs have a single subregion), the model parameters are not well defined and require special handling. At the end, the maps produced by this alternative procedure were essentially the same as those obtained by the non-parameteric methods described above, which are about 1000 times faster to compute. We have verified that the various relationships described in preliminary results, will hold under other reasonable measures of these same quantities.
